# Phagocytic ability of neutrophils and monocytes in neonates

**DOI:** 10.1186/1471-2431-11-29

**Published:** 2011-04-14

**Authors:** Athanasios Filias, Georgios L Theodorou, Sofia Mouzopoulou, Anastasia A Varvarigou, Stephanos Mantagos, Marina Karakantza

**Affiliations:** 1 Paediatric Department Medical School & University Hospital, University of Patras, Patras, Greece; 2 Division of Hematology, Department of Internal Medicine, Medical School & University Hospital, University of Patras, Patras, Greece

**Keywords:** Cord blood, Escherichia Coli, Monocytes, Neonate, Neutrophils, Phagocytosis, Phagocytic ability

## Abstract

**Background:**

Infections by a variety of pathogens are a significant cause of morbidity and mortality during perinatal period. The susceptibility of neonates to bacterial infections has been attributed to immaturity of innate immunity. It is considered that one of the impaired mechanisms is the phagocytic function of neutrophils and monocytes. The purpose of the present study was to investigate the phagocytic ability of neonates at birth.

**Methods:**

The phagocytic ability of neutrophils and monocytes of 42 neonates was determined using the Phagotest flow cytometry method, that assesses the intake of *E. Coli *by phagocytes, in cord blood and in peripheral blood 3 days after birth. Fifteen healthy adults were included in the study as controls.

**Results:**

The phagocytic ability of neutrophils in the cord blood of neonates was significantly reduced compared to adults. The 3^rd ^postnatal day the reduction of phagocytic ability of neutrophils was no longer significant compared to adults. The phagocytic ability of monocytes did not show any difference from that of adults either at birth or the 3^rd ^postnatal day.

**Conclusions:**

Our findings indicate that the intake of *E. Coli *by phagocytes is impaired at birth in both preterm and full term neonates compared to adults. This defect is transient, with the phagocytic ability in neonates reaching that of the adults 3 days after birth.

## Background

Infections by a variety of pathogens are a significant cause of morbidity and mortality during perinatal period. The incidence of early-onset sepsis in full term neonates is 0.1% while in premature ones is as high as 0.4% [1]. In a prospective study in seven Australian Neonatal Intensive Care Units (NICUs), Isaacs et al [2] reported an annual incidence of sepsis of 6.6 per 1000 live births, of which 75% were of late onset. Overall hospital mortality for sepsis was 10% [2]. In a cohort of 54 UK neonatal units in 1998, 204 (5%) of 3,963 consecutive admissions had a positive blood culture [3]. In a North American cohort, mortality in very low birth weight infants with septicemia was 21% [4]. The increased susceptibility of neonates to bacterial infections has been attributed to immaturity of innate immunity.

The phagocyte system is an essential component of innate immunity, where specialized phagocytes (macrophages, monocytes and neutrophils) perform various host defense functions that rely on the phagocytic uptake of pathogens. A number of factors contribute to the efficient function of phagocytic system. These factors include the presence of adequate numbers of monocytes and neutrophils in the peripheral blood, the ability to respond to signals from sites of inflammation, the migration to these sites and the capacity to ingest and kill the invaded microorganisms.

Multiple subtle deficiencies of the phagocytic system have been described in neonates. Neonates have decreased bone marrow neutrophil storage pool, resulting in production of inadequate numbers of neutrophils in response to bacterial sepsis [5,6]. Cord blood phagocytes show also decreased chemotactic response. This has been attributed to reduced expression of adhesion molecules involved in migration as Mac-1 and L-selectin [7-10].

Many of the functions of phagocytic system are cytokine inducible. The production of cytokines that activate monocytes and neutrophils in neonates is impaired compared to adults [5]. The protein production, the mRNA expression as well as the half life of mRNA of GM-CSF, G-CSF and M-CSF are also significantly decreased in cord blood compared to adults [5,11]. In addition, there are reports showing that in neonates the phagocytic activity of neutrophils and monocytes, per se, is defective [12-14]. Decreased intake of bacteria might be due to reduced levels of IgG antibodies and complement proteins resulting in impaired opsonization and/or reduced expression of relevant receptors [9,15-21]. The bacterial killing might be reduced even when their intake is normal, due to decreased intracellular production of oxidative radicals [17,22].

The published data on phagocytosis and intracellular bacteria killing are difficult to be interpreted due to the many parameters involved as well as the variety of techniques used in the last 30 years for the investigations of these aspects of neutrophils function. Recent reports using flow cytometry techniques confirm the defects on aspects of intracellular killing reported by previous techniques, but they do not add information on the phagocytic activity of neonatal neutrophils [22,23].

The purpose of the present study was to investigate the phagocytic ability of neonatal neutrophils and monocytes in cord blood and in peripheral blood 3 days after birth.

## Methods

### Participants

The study was performed at the Division of Neonatology of the Department of Pediatrics, at the General University Hospital of Patras (Rio, Greece), from January 2007 to March 2007. A cohort of 42 neonates was enrolled in the study. Twenty of the 42 neonates were males and 22 females. Seven were premature (32 - 37 weeks of gestation) and 35 were full term neonates. Twenty one of the 42 neonates delivered vaginaly and 21 by elective cesarean section.

Umbilical mixed arterial-venous blood was collected aseptically immediately after birth. Venous blood was drawn the 3^rd ^day of life in all study neonates. All samples were collected in tubes containing heparin.

Neonates admitted to the study were singleton, had intact membranes, clear amniotic fluid, an Apgar score equal to or greater than 7 at 1 and 5 minutes, and no signs of distress or infection during intrauterine life or evident of congenital anomalies. The mothers of the neonates were healthy, and receiving no medications. Their present pregnancy was uneventful with no history of preeclampsia, diabetes or hypertension, and did not receive steroids. During the first three days of life none of the neonates developed signs of infection, as defined by clinical status, white blood cells count, and C-reactive protein levels. Randomly selected unrelated healthy adults donated blood and served as controls (8 males, 7 females, median age 30, and range: 22-36 years).

The ethical rules of the Declaration of Helsinski for experimentation with humans were strictly observed throughout this investigation. The study was conducted under the auspices of the Ethical Committee of the University Hospital of Patras (Rio, Greece), and an informed consent was obtained from the parents of the newborns and the volunteer adults before enter of the study.

### Phagocytic assay

The phagocytic activity, was investigated in cord blood and in peripheral blood the 3rd day after birth. In vitro phagocytic activity, was determined using the Phagotest kit (Opregen Pharma, Heidelberg, Germany). The principle of the test is that whole blood is incubated with opsonised (by complement and immunoglobulin) *E. Coli *that are labelled by fluorescein (FITC). Bacteria are ingested by phagocytes generating a green fluorencence signal, quantified by flow cytometry [24]. The test was performed according to the instructions of the provider. Briefly, 100 μl heparinized whole blood was incubated with FITC-labelled *E. Coli *(2 × 10^7 ^per 20 μl) at 37°C for 10 minutes and in parallel a negative control sample remain on ice. The amount of bacteria added in each sample, was calculated in order the ratio of bacteria to leucocytes to be 25:1. At the end of the incubation phagocytosis was stopped by placing the samples on ice. To eliminate the fluorescence of non-phagocytized bacteria, 100 μl of quenching solution were added. The cells were washed twice with 3 ml washing solution (5 min, 250 × g, 4°C). Cells then were re-suspended and incubated for 20 min in 2 ml lysing solution for lysis of erythrocytes and fixation of the leucocytes. After a final wash with 3 ml of washing solution (5 min, 250 × g, 4°C), the cells were re-suspended in 200 μl of DNA staining solution to excludes aggregation artifacts of bacteria or cells, and analyzed by flow cytometry as we describe below.

### Flow cytometric analysis

Samples were analyzed by flow cytometry using a Coulter EPICS-XL-MCL cytometer, and the data were processed using the XL-2 software (Coulter, Miami, Florida, USA). During data acquisition a gate was set in the red fluorescence histogram on those events which had the same DNA content as a human diploid cell, in order to exclude extracellular bacteria. Dead cells were excluded in the FCS vs SSC scatter diagram. The phagocytic ability was evaluated in neutrophils and monocytes. Live populations were gated by the software program in the scatter diagram (FCS vs SSC) and their green fluorescence histogram (FL1) was analyzed. We collected 10.000-15.000 leukocytes per sample.

The phagocytic ability was expressed as percentage of fluorescent cells in the total population studied and calculated by subtracting the percentage of the negative control sample (<1%) from the positive sample. The intra-assay coefficient variation for neutrophil phagocytosis is <2% while for monocyte phagocytosis is <5%.

### Statistical analysis

The proportions between term and preterm neonates (gender, gestational age, mode of delivery) were compared with Fisher's exact test.

Data analyses were performed by applying parametric and non parametric tests, as appropriate. Values were expressed as mean with standard deviation or median with interquartile range. For the comparisons between phagocytic ability in the cord blood and third day of life of the newborns the paired *t *test and the Wilcoxon test were used. In order to compare the values of the neonates' cord blood or the third day and the adults the unpaired *t *test and the Mann-Whitney was applied, as required. The phagocytic ability and the absolute number (cells/μL) of neutrophils and monocytes, were correlated to birth weight and gestational age by Spearman rank correlation. A P value of <0.05 was considered significant. All statistical analyses were performed with SPSS V17.0 software (SPSS, Chicago, IL, USA).

## Results

The sex, gestational age, mode of delivery, birth weight and the size for gestational age of preterm and term neonates are shown in Table 1. The proportions between term and preterm neonates (gender, size for gestational age, mode of delivery) were not statistically different as we have shown with Fisher's exact test (Table 1).

**Table 1:** Characteristics of the neonates studied

	PRETERM	TERM	*P*
Number	7	35	
Male Sex	5	15	0.23 *
Gestational age (mean ± SD; weeks)	35.3 ± 1.2	38.7 ± 1.1	0.00005 **
Birth Weight (mean ± SD; g)	2350 ± 502	3261 ± 347	0.001 **
Size for Gestational Age (LGA/AGA)	2/5	5/30	0.58 *
Delivery (Vaginal/Cesarean)	2/5	19/16	0.41 *

LGA: Large for gestational age, AGA: Appropriate for gestational age;

*Fisher's exact test

** T-test

The absolute number of neutrophils and monocytes were significantly increased in the cord blood compared to adults, *P *= 0.006 and *P *= 0.004 respectively. The number of cord blood neutrophils and monocytes were reduced on the third day of life but remained significantly higher compared to that of adults, *P *= 0.007 and *P *= 0.02 respectively (Table 2).

**Table 2:** Phagocytic ability of neutrophils and monocytes in the cord blood and at the 3^rd ^day of life in comparison to adult controls.

	Neonates (n = 42)			Significance (*P *value)		
			
	Cord Blood	Day 3	Adults (n = 15)	Cord Blood - day 3	Cord Blood adults	Day 3 - adults
Absolute Number of Neutrophils (cells/μL)	6750 (5054-8800)	4765 (3930-6050)	3200 (2860-3780)	**0.003**	**0.006**	**0.007**
Phagocytic ability of neutrophils (%)	70.4 (55.8-81.7)	75.6 (66.6-85.7)	77.0 (74.8-81.2)	0.236	**0.012**	0.45
Absolute Number of Monocytes (cells/μL)	1100 (800-1415)	1000 (522-1650)	500 (430-650)	0.844	**0.004**	**0.02**
Phagocytic ability of Monocytes (%)	59.8 (39-66.2)	60.5 (45.3-66.6)	57.1 (49.7-61.9)	0.477	0.679	0.871

Data are expressed as medians and interquartile ranges (in parentheses), and were tested for significance with the Mann-Whitney U test. NS statistically non-significant difference

### Phagocytic ability

The phagocytic ability of neutrophils in the cord blood was significantly reduced compared to that of adults, *P *= 0.012. This impairment was not noted when the neutrophils of cord blood from preterm and full term neonates were compared separately with adults, probably due to the small number of individuals included in each group.

In the 3^rd ^postnatal day the phagocytic ability of neutrophils increased compared to that in cord blood and did not differ significantly from that of adults (Table 2). The phagocytic ability of monocytes in the cord blood and the 3^rd ^postnatal day did not differ from that of adults (Table 2).

**Figure 1:**
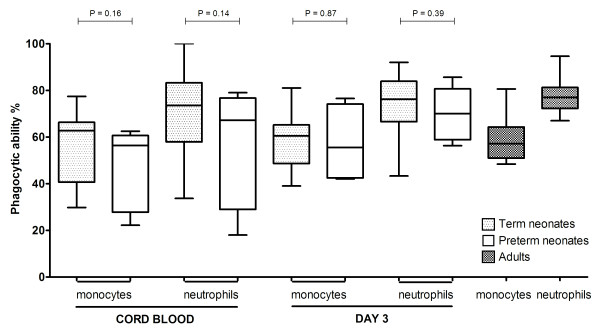
**Comparison of the phagocytic ability of neutrophils and monocytes in the cord blood as well on the third day of life between preterm and full term neonates**. Comparisons between groups revealed no statistically significant differences. The phagocytic ability of adult neutrophils and monocytes is also shown.

The phagocytic ability of neutrophils and monocytes in the cord blood as well as the 3^rd ^postnatal day did not differ between those born by vaginal delivery and cesarean section as well as among female and male neonates (*P *> 0.05). Preterm neonates showed a lower phagocytic ability of neutrophils and monocytes in the cord blood as well as the 3^rd ^postnatal day than the full term neonates; however, these differences were not statistically significant (Figure 1).

**Figure 2:**
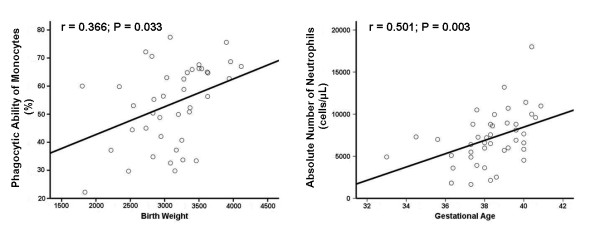
**Correlations of phagocytic ability of monocytes with birth weight, and absolute number of neutrophils with gestational age of the neonates**.

Spearman rank correlation showed a significant relation of birth weight with the phagocytic ability of monocytes (r = 0.366, *P *= 0.033) and of gestational age with the absolute number of the neutrophils (r = 0.501, *P *= 0.003) (Figure 2). It did not show any significant correlation between gestation age and monocyte and neutrophil phagocytosis. This finding might be due to the small number of preterm neonates included in the study and the fact that all of them were late preterm neonates.

## Discussion

In this study we investigate the phagocytic ability of neonatal neutrophils and monocytes in cord and peripheral blood 3 days after birth. The study demonstrates that the percentage of neutrophils in cord blood that have the ability to phagocytose *E. Coli *is reduced in neonates compared to healthy adults. This finding is transient and the neonatal phagocytic ability reaches that of healthy adults 3 days after birth.

The observed differences in absolute numbers of monocytes and neutrophils between cord blood and peripheral blood 3 days after birth are expected. The homeostasis of neutrophil and monocyte pools the first days after birth is basically unknown. Many factors might participate in the regulation of homeostasis as production of cytokines, decline of hormones, maturation of macrophages, substances transferred by breastfeeding as soluble CD14 and others. Some of these factors might have an effect on the cellular function and account for the increase in the phagocytic ability noted in our study [25].

Previous studies performed mainly in the '80s using techniques that assessed phagocytosis on separated mononuclear cells with the addition of adult and/or neonatal serum concluded that the intake of bacteria is impaired in neonates [10,12,17,18]. The intake of bacteria is compromised in neonates either due to decreased levels of opsonized proteins or/and to low levels of receptors involved in phagocytosis [6,10]. Numerous studies have shown that complement components are reduced in both premature and full term neonates [15,16,26-28]. The levels of IgG immunoglobulin's in neonatal serum are proportional to gestational age reaching concentrations similar or even in excess to that of maternal serum in term neonates [16,29]. However, there are data showing that some IgG subtypes are less efficiently transferred across the placenta while the capacity of transplacental derived maternal IgG to activate complement varies widely [16].

In neonates the levels of CR1 complement receptors and of FcRII immunoglobulin receptors are similar to that of adults [30,31]. However the expression of the complement receptor CR3 has been found reduced on monocytes and neutrophils at birth, reaching those of adults at the age of 2 weeks for full term neonates to over 2 months for premature infants [7,30,32,33]. In preterm neonates before 32 weeks the expression of the immunoglobulin receptor FcRIII is reduced to <50% of adult levels reaching normal levels two weeks after birth [30,34].

The *in vitro *method used in our study for evaluation of phagocytic ability assesses by flow cytometry the mechanisms involved in the intake of opsonized by complement and immunoglobulin *E. Coli *in whole blood [24]. The defects therefore detected in the study do not reflect reduced levels of opsonised proteins in the plasma of neonates but demonstrate reduction of receptors significant for the intake of *E. Coli*. These include receptors not only recognizing opsonised bacteria such as CR1 and FcRII but also receptors recognizing directly bacterial structures as CR3, FcRIII and Toll-like receptors (TLRs) [6]. Our findings regarding neutrophils confirm those of a previous study evaluating phagocytosis by the same method as ours that had shown that the phagocytic ability of neutrophils is reduced in cord blood compared to normal adults [13]. Strunk et al. [13] have shown that this impairment depends on age as fetuses are more severely affected than full term neonates. However we did not find a phagocytic defect on neonatal monocytes, a finding differing from those of previous studies that have report a similar defect in both populations. This discrepancy might be due to differences in study populations, as previous studies included neonates with smaller gestational age than ours [13,35].

Effective phagocytosis requires, apart from bacteria intake, intracellular processes resulting in bacteria degradation. There are reports in the literature showing that oxidative burst is reduced in neonatal phagocytes suggesting that even if the mechanisms involved in the bacteria intake are intact the bacteria degradation is not effective [17,22]. However a recent study investigating the production of reactive oxygen species (ROS) after *E. Coli *and group B *Staphyloccoci *(GBS) intake, in a similar in vitro model as ours, has not found a difference between the ROS production in neonatal and adult phagocytes [36]. Evenmore, the study showed that after bacteria challenge the phagocytosis induced cell death (PICD) is reduced in cord blood compared to adult peripheral blood. The non apoptotic leucocytes can result in activation of immune system and overwhelming production of cytokines [37]. Recently it has been shown that reduced monocyte apoptosis during the initial phase of sepsis is related to poor outcome in adults [38]. In neonates, the decrease of PICD might be a mechanism that initiates a fulminant hyper-inflammation response resulting in sepsis.

The transient impairment of *E. Coli *intake by phagocytes did not have clinical significance for the neonates included in the study. All neonates in the present study were healthy and did not develop infectious complications. The elevated absolute numbers of neutrophils and monocytes noted in neonates might partially compensate the defect found in the ingestion of bacteria by these cells at birth. However, our study was not designed to address clinical issues. Prospective studies are needed in order to correlate in vitro findings with clinical parameters.

## Conclusion

In conclusion, our study demonstrated that intake of *E. Coli *by phagocytes is impaired in neonates compared to adults. The 3^rd ^day after birth the phagocytic ability of neonates reached that of adults. Clinical studies are needed to clarify the impact that this transient impairment has to neonatal susceptibility to bacterial infections and their overall outcome.

## Competing interests

The authors declare that they have no competing interests.

## Authors' contributions

AF collected the material for the study, performed the testing of samples, analyzed the results and participated in the preparation of the manuscript. GLT supervised the flow cytometry testing, analyzed the flow cytometry results, participated in the statistic analysis of the results and the preparation of the manuscript. SM^2 ^participated in the testing of samples. AAV participated in the design of the study, the collection of material and revised the manuscript. SM^1 ^participated in the design of the study. MK participated in the design of the study and preparation of the manuscript. All authors read and approved the final manuscript.

## Pre-publication history

The pre-publication history for this paper can be accessed here:

http://www.biomedcentral.com/1471-2431/11/29/prepub
